# Inhibition of LINK-A lncRNA overcomes ibrutinib resistance in mantle cell lymphoma by regulating Akt/Bcl2 pathway

**DOI:** 10.7717/peerj.12571

**Published:** 2021-12-17

**Authors:** Ye Zhang, Peng Lu, Yan Zhou, Lifei Zhang

**Affiliations:** 1 Department of Hematology, Sir Run Run Shaw Hospital, Zhejiang University School of Medicine, Hangzhou, Zhejiang, China; 2 Department of Neurosurgery, Sir Run Run Shaw Hospital, Zhejiang University School of Medicine, Hangzhou, Zhejiang, China

**Keywords:** LINK-A, Ibrutinib, Mantle cell lymphoma, AKT, Drug resistance

## Abstract

Ibrutinib, a bruton tyrosine kinase (BTK) inhibitor which suppresses B-cell receptor signaling, has remarkably improved the outcome of patients with mantle cell lymphoma (MCL). However, approximately 33% of MCL patients have primary Ibrutinib resistance, and acquired Ibrutinib resistance is nearly universal. Long intergenic non-coding RNA for kinase activation (LINK-A) exerts oncogenic role in different types of tumors, but the role of LINK-A in intrinsic ibrutinib resistance in MCL is still unclear. Here, LINK-A expression level was first assessed using quantitative Real-time PCR (qPCR) and immunofluorescence analysis in five MCL cell lines. The effect of LINK-A on regulating MCL cells viability and apoptosis was assayed using CCK-8 and TdT-mediated dUTP nick end labeling (TUNEL) assay, respectively. The association of LINK-A with AKT activation and B cell lymphoma 2 (Bcl2)expression was evaluated using qPCR and western blot analysis. We found that LINK-A level was elevated in Ibrutinib-resistant MCL cell lines (Mino, REC-1, MAVER-1, and Granta-519) compared to Ibrutinib-sensitive MCL cell lines (Jeko-1). Functionally, LINK-A overexpression in Jeko-1 cells enhanced cell viability and repressed Ibrutinib-induced cell apoptosis. LINK-A knockdown in MAVER-1 cells decreased cell viability and further accelerated Ibrutinib-induced cell apoptosis. LINK-A overexpression enhanced Bcl2 expression in Jeko-1 cells, and Bcl2 inhibition blocked the effect of LINK-A on increasing cell viability in the presence of Ibrutinib. On the contrary, LINK-A knockdown reduced Bcl2 expression in MAVER-1 cells, and Bcl2 overexpression damaged the role of LINK-A inhibition in regulating cell viability. Mechanistically, LINK-A positively regulated the activation of AKT signaling, and inhibition of AKT signaling destroyed LINK-A-induced increased of Bcl2 and resulted in a subsequent suppression of cell viability. Taken together, the current results demonstrate that LINK-A inhibition overcomes Ibrutinib resistance in MCL cells by regulating AKT/Bcl2 pathway.

## Introduction

Mantle cell lymphoma (MCL) is a highly aggressive non-Hodgkin’ lymphoma originating from B cells, accounting for 6% of all cases of this disease (Gujral et al., 2009; Lian et al., 2009). The incidence rate of MCL is increasing year by year, and it seriously threatens human health (Cheah, Seymour & Wang, 2016). In recent years, the diagnosis and therapy of MCL have made some progress, but there is still no standard treatment (Jain et al., 2019; Klener, 2019) Although immune targeted therapy combined with high-dose chemotherapy improves the remission rate of patients, many patients remain non-responsive (Lazar, 2009; Chen, Teo & McCarty, 2016). Therefore, new drugs and new treatment strategies are urgently needed.

As the first effective and highly selective Bruton’s tyrosine kinase (BTK) inhibitor, Ibrutinib irreversibly inhibits the activity of BTK by selectively covalently binding to the cysteine residue binding site (Cys-481) of the target protein BTK (Buggy & Elias, 2012; Singh, Dammeijer & Hendriks, 2018). BTK plays a crucial role in the survival, development, differentiation, proliferation, adhesion and migration of B cells (Burger, 2019). After activation, BTK can activate NF- *κ*B and RAS/Raf/MEK/ERK signaling pathways, both of which are involved in the development of B cells (Roskoski Jr, 2016). The B cell antigen receptor (BCR) signal is abnormally active in B-cell tumors, which includes BTK (Koehrer & Burger, 2016). Ibrutinib can inhibit BTK irreversibly, thus blocking the abnormal signal transduction in B-cell tumor, and finally play an anti-B-cell tumor role (Advani et al., 2013). Preclinical studies have shown that Ibrutinib can induce MCL apoptosis by inhibiting BCR signal transduction (Cinar et al., 2013). Although Ibrutinib treatment has improved markedly the prognosis of patients with MCL, approximately one-third of the patients developed drug resistance. Revealing the underlying mechanism of Ibrutinib resistance is indispensable to improving the treatment of MCL.

Long noncoding RNA (lncRNA) is a kind of non-coding RNA with a length of more than 200 nt (Jathar et al., 2017; Xu et al., 2017). The discovery of lncRNA provides an important opportunity for studying the biological behavior of malignant tumors and revealing the nature of chemotherapy resistance (Wang et al., 2018; Liu et al., 2018), LncRNA LINK-A, a long intergenic non-coding RNA for kinase activation, is involved in breast cancer resistance and hypoxia (Lin et al., 2016a; Lin et al., 2017a). However, the role of LINK-A in the resistance of MCL to ibrutinib and its underlying mechanism are not clear.

Here, we investigated the LINK-A expression in Ibrutinib-sensitive MCL cell lines and Ibrutinib-resistant MCL cell lines. Also, we explored the effect of LINK-A on regulating MCL cells viability and apoptosis, and further investigate the association of LINK-A with AKT activation and Bcl2 expression. Taken together, all results demonstrate that LINK-A inhibition contributes to overcome Ibrutinib resistance of MCL cells by regulating the AKT/Bcl2 pathway.

## Materials and Methods

### Cell culture

Ibrutinib-resistant MCL cell lines (MAVER-1 and Granta-519), Ibrutinib-intermediated MCL cell lines (Mino and REC-1), and Ibrutinib-sensitive MCL cell lines (Jeko-1) were procured from the ATCC (Manassas, VA, USA). These cells were kept in RPMI-1640 (GIBCO, Grand Island, NY, USA) contained with 10% FBS in the humidified 5% CO_2_ environment at 37 °C. 5 ×10^4^ Jeko-1 and MAVER-1 cells were incubated with Ibrutinib (0.6 µmol/L; Selleck, TX, USA) for 48 h.

### RNA interference (RNAi) and overexpression

For overexpression experiments, Jeko-1 and MAVER-1 cells were transfected with the pcDNA3 plasmid and recombinant plasmids (pcDNA3-LINK-A and pcDNA3-Bcl2, constructed in our lab) as instructed by the manufacturer for Lipofectamine 2000 (Invitrogen) 48 h. For knockdown experiments, Jeko-1 and MAVER-1 cells were transfected with the indicated siRNA as instructed by the manufacturer for Lipofectamine RNAiMAX (Thermo Fisher Scientific) 48 h. All siRNAs (non-targeting control siRNA, NC-siRNA; siRNAs targeting LINK-A, and Bcl2) were obtained from HANBIO (Shanghai, China).

### Western blot

Protein was extracted from Jeko-1 and MAVER-1 cells by RIPA Lysis Buffer (Sangon, Shanghai, China). The protein quantification was detected with BCA Protein Quantification Kit (Vazyme, Nanjing, China). After that, protein samples were separated by 12% SDS-PAGE, and transferred to PVDF membranes (Roche, Basel, Switzerland). The membranes were washed with 5% nonfat milk in TBST, and incubated with primary antibodies for 4 °C overnight. The following primary antibodies were used: anti-Bcl2 (Abcam, ab194583, 1/2000), anti-Akt (Abcam, ab18785; 2 µg/ml), anti-Akt (phospho, Abcam, ab38449, 1/1000), and anti- *β*-actin (Abcam, ab7817; 1:2000). The membranes were incubated with HRP-conjugated secondary anti-rabbit (1:5000, Abcam) for 60 min at room temperature (RT). Immunoreactive bands were visualized with the ECL detection system (GE Healthcare, IL, USA). The intensity of protein bands was quantified by ImageJ software.

### Fluorescence *in situ* hybridization analysis (FISH)

The subcellular localization of LINK-A was detected via FISH with RiboTM lncRNA FISH Probe Mix (Green) (Ribo Biotech, China). Jeko-1 and MAVER-1 cells (2. 5 ×10^4^/well) were mounted onto slides and fixed in formaldehyde (4%) for 15 min. The slide was pretreated with protease K (2 µg/mL), glycine and acetic anhydride, and pre-hybridization for 60 min and hybridization at 42 °C with probes (250 µL, 300 ng/mL) against LINK-A. Lastly, the slide was stained using phosphate buffered saline with DAPI (Sigma-Aldrich, St. Louis, MO, USA).

### TUNEL assay

TUNEL assay was performed to observe cell apoptosis. Briefly, Jeko-1 and MAVER-1 cells were treated with Ibrutinib (0.6 µmol/L) for 48 h, MK2206 (3 Mm, Selleck, TX, USA) for 24 h or transfected with LINK-A overexpression or knockdown for 48 h and fixed in 4% formaldehyde. The cells were stained using the TUNEL kit as the manufacturer’s instructions (Roche). TUNEL-positive cells were observed with fluorescence microscopy (DMI4000B, Leica).

### RNA extraction and reverse transcriptase quantitative real-time PCR (qPCR)

Total RNA was extracted from Jeko-1 and MAVER-1 cells with Trizol reagent (Sangon Biotech, Shanghai, China) according to the manufacturer. Total RNA was reverse transcribed to cDNA by cDNA Reverse Transcription Kit (Takara, Japan). qPCR was carried out on ABI 7500-Fast Real-Time PCR System (Applied Biosystem, Foster City, CA, USA) with ChamQ SYBR qPCR Master Mix (Vazyme). The qPCR primers used in the study were listed in Table 1. The fold changes of RNA transcripts were calculated using the 2^−ΔΔCt^ method, and *β*-actin was used as a control gene.

### Cell vitality

Cell Counting Kit-8 (CCK-8) assay was performed to determine cell viability. Jeko-1 and MAVER-1 cells were seeded in 96-well plates (4 ×10^3^ cells per well) and treated with Ibrutinib (0.6 µmol/L) for 48 h, MK2206 (3 µM) for 24 h or transfected with LINK-A overexpression or knockdown for 48 h. After that, the 10 µL CCK-8 solution was added for another 2 h at 37 °C. The absorbance was measured at 450 nm with a microplate reader (Thermo Fisher Scientific, Waltham, MA, USA).

### Statistical analysis

Data are expressed as mean ± Standard Deviation (SD). All statistics were performed with SPSS 16.0 (SPSS Incorporation, Chicago, IL). The difference between two groups was compared by two-tailed student’s *t*-test, or one-way analysis of variance (ANOVA) followed by the Scheffé test. *P*-values < 0.05 were considered statistically significant.

## Results

### LINK-A expression was elevated in Ibrutinib-resistant MCL cell lines

**Figure 1:**
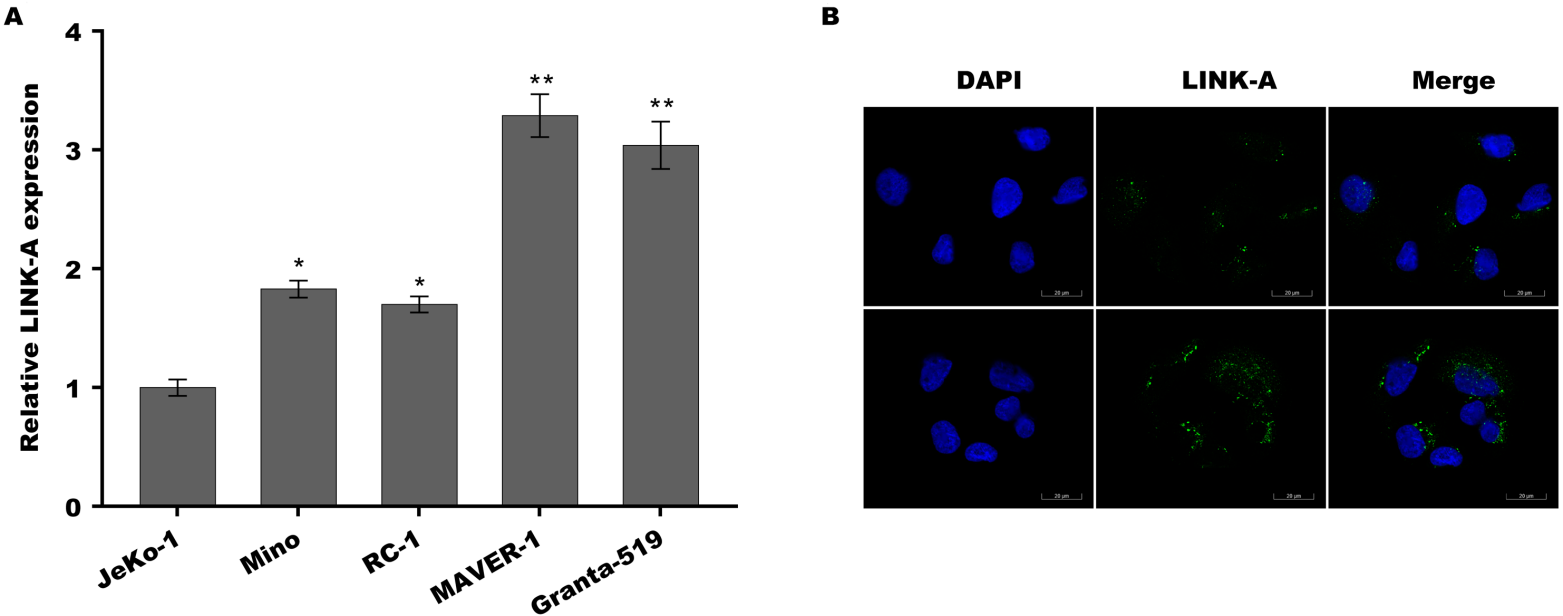
LINK-A expression was elevated in Ibrutinib-resistant MCL cell lines.

Previous studies have demonstrated that Jeko-1 is Ibrutinib-sensitive MCL cell lines, Mino and REC-1 are Ibrutinib-intermediated MCL cell lines, and MAVER-1 and Granta-519 are Ibrutinib-resistant MCL cell lines (Ming et al., 2018; Rahal et al., 2014; Ma et al., 2014). To investigate the role of LINK-A in MCL progression and Ibrutinib resistance, the expression of LINK-A was assessed in these differential ibrutinib resistant MCL cell lines using qPCR and immunofluorescence analysis. Figure 1A showed that LINK-A expression level was higher in Mino and REC-1 cells than in Jeko-1 cells, and was further increased in MAVER-1 and Granta-519 cells. The results from immunofluorescence analysis also showed that LINK-A expression level was higher in MAVER-1 cells than in Jeko-1 cells (Fig. 1B). These results indicate that upregulated LINK-A might be correlated with intrinsic Ibrutinib resistance in MCL.

**Table 1:** qPCR primers used in the study.

Genes	Sense (5′–3′)	Antisense (5′–3′)
LINK-A	GATGACATTTGTAGCTGGGAGC	TGAAGCAGGGTTTTATTGGGTG
Bcl2	TTCGGTGGGGTCATGTGTGTG	GTGTGCAGGTGCCGGTTCAG
Toso	GAACACAGACCGGGGAAAGAC	ACAAATAGGGCAGATGAAACCAT
PIK3CD	GGCTTCTCTTCCTCCACCTCTT	CACTTTGGTTTTCCAGCTCTCAC
IKBKB	CTTGGGACAGGGGGATTTGG	CTCAGGGACATCTCGGGCAG
TNFRSF7	GCAGTGCAGGGACAAGGAGTG	AGGTAAGTGGGTGGGCTGAGG
CASP7	AATTTATGGGAAAGATGGTGTCAC	CCTGAATGAAGAAGAGTTTGGGT
STK17A	TATGAGACTGCATCAGAAATGATC	CACGAGTGTGTAAAAAGTGAACACC
CSE1L	TCCCCTACATCCCTACTCTCATC	TAAACACCAAAAACAAAGCCTCC
STK17B	TCTAAAAAAGAGAAGAAGAGGACAGG	TTCAGCCAACTCAGGTAAACACAG
BIRC5	TCCACTGCCCCACTGAGAAC	GAAAGCGCAACCGGACGAAT
Beta-actin	GGCATCCACGAAACTACATTCAA	AGCCAGAGCAGTGATCTCCTTCT

### LINK-A enhanced cell viability and repressed Ibrutinib-induced cell apoptosis

**Figure 2:**
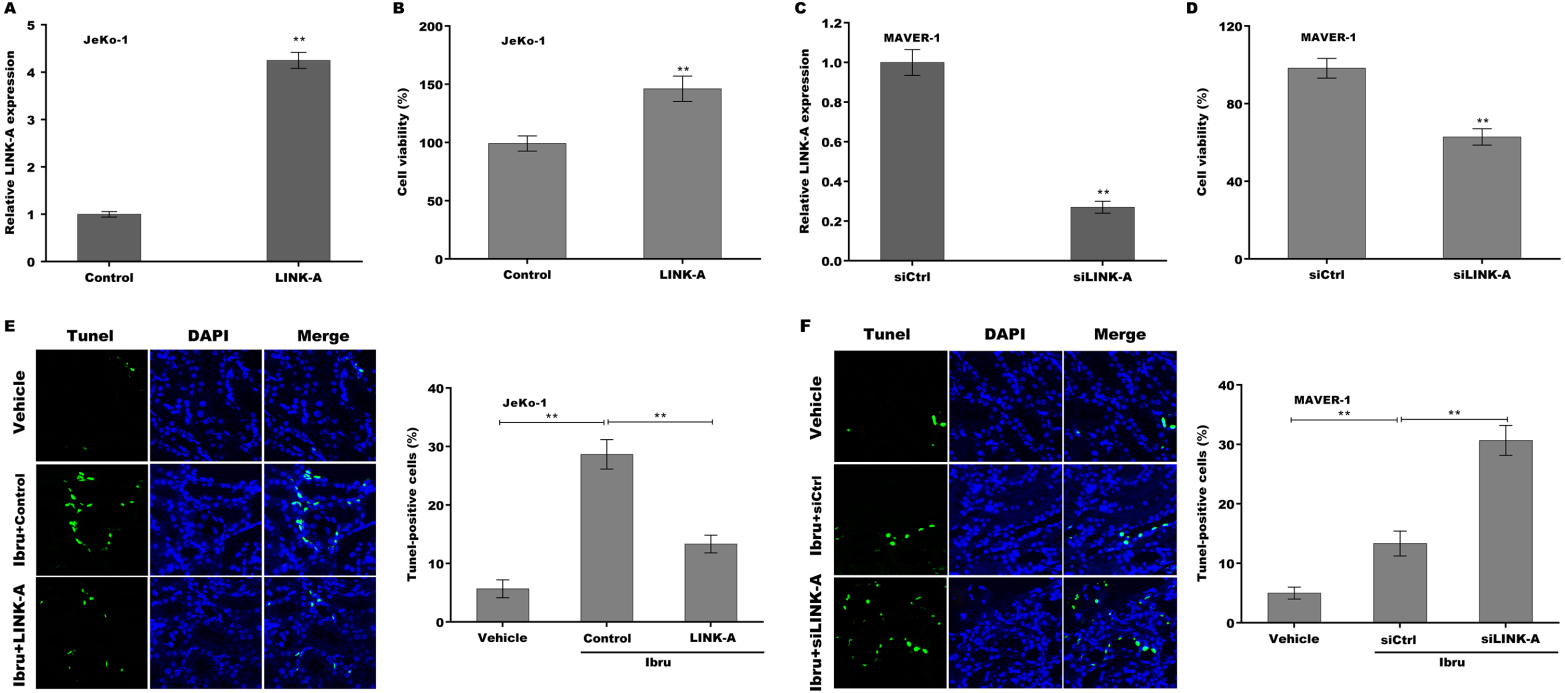
(A–F) LINK-A enhanced cell viability and repressed Ibrutinib-induced cell apoptosis.

To explore the function of LINK-A on regulating cell viability and Ibrutinib-induced cell apoptosis, LINK-A was overexpressed in Jeko-1 cells or knocked down in MAVER-1 cells and then cell viability and apoptosis were assessed. As shown in Figs. 2A–2D, LINK-A overexpression significantly enhanced Jeko-1 cell viability, whereas knockdown of LINK-A repressed MAVER-1 cell viability. Furthermore, the results from TUNEL assay showed that Ibrutinib treatment resulted in a marked increase of Jeko-1 cell apoptosis, whereas LINK-A overexpression weakened remarkably the effect of Ibrutinib on facilitating Jeko-1 cell apoptosis (Fig. 2E). In MAVER-1 cells, knockdown of LINK-A further promoted Ibrutinib-induced cell apoptosis (Fig. 2F).

### LINK-A enhanced Bcl2 expression to increase cell viability in the presence of Ibrutinib

**Figure 3:**
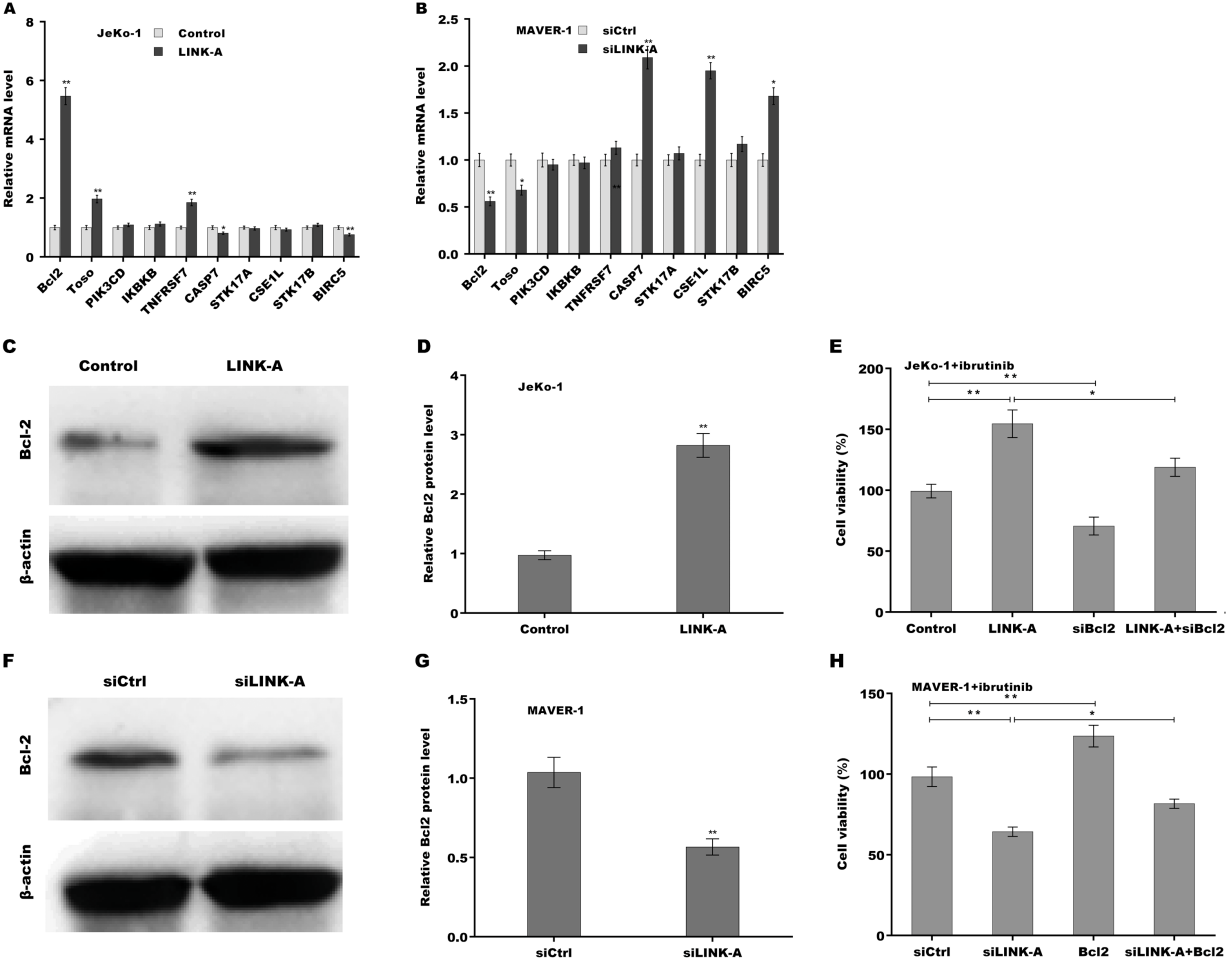
(A–H) LINK-A enhanced Bcl2 expression to increase cell viability in the presence of Ibrutinib.

Given the important role of LINK-A in regulating cell apoptosis, the target genes of LINK-A in the apoptosis pathway were next investigated. An integrated analysis based on a cDNA microarray (containing 6386 cancer-related genes) revealed that 23 apoptosis-related genes are dys-regulated in most samples (Table S1) (Martinez et al., 2003). Here the correlation of LINK-A with the top five up-regulated and top five down-regulated genes was assessed in MCL cells. Figure 3A showed that LINK-A overexpression resulted in the increase of Bcl2, Regulator of Fas-induced apoptosis (Toso), and Tumor necrosis factor receptor superfamily, member 7 (TNFRSF7), and the decrease of Caspase 7 and survivin in Jeko-1 cells. LINK-A knockdown caused the decreased expression of Bcl2 and Toso, and the increased expression of Caspase 7, CSE1L and survivin in MAVER-1 cells (Fig. 3B). Given the crucial role of Bcl2 in BTK activation and MCL progression (Li et al., 2016; Agarwal et al., 2018), we further explored the role LINK-A in regulating Bcl2 expression in MCL cell lines. Figures 3C and 3D showed that LINK-A overexpression enhanced Bcl2 protein expression in Jeko-1 cells, whereas knockdown of LINK-A repressed Bcl2 protein expression in MAVER-1 cells (Figs. 3F and 3G). Functionally, the results from CCK-8 assay showed that Bcl2 inhibition weakened the effect of LINK-A on facilitating cells viability (Figs. S1A and S1B, Fig. 3E), whereas Bcl2 overexpression weakened the effect of LINK-A inhibition on regulating cells viability (Figs. S1C and S1D, Fig. 3H). These data demonstrate that LINK-A increases cell viability by enhancing Bcl2 expression in the presence of Ibrutinib.

### LINK-A increased Bcl2 expression by activating AKT signaling

**Figure 4:**
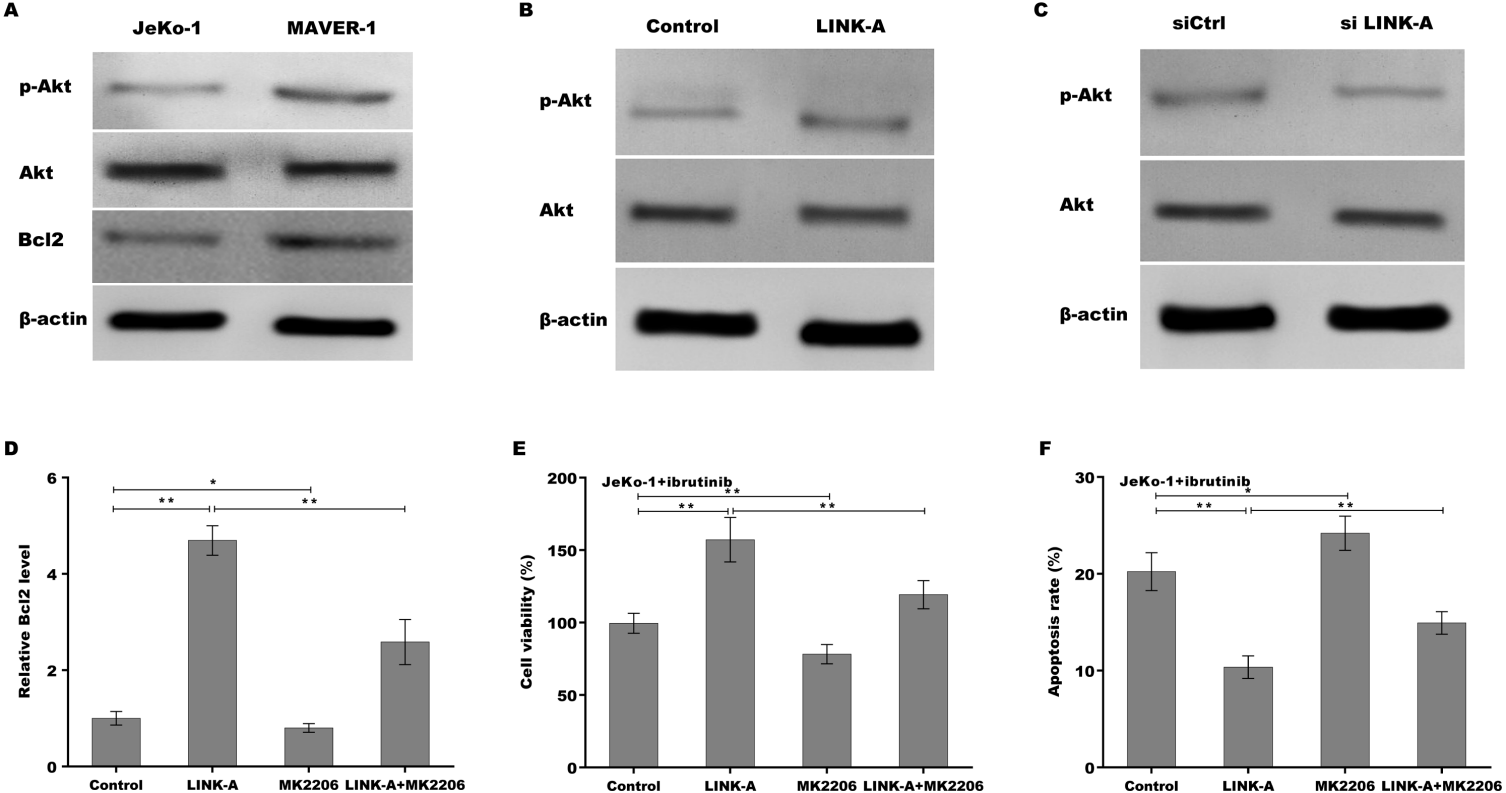
(A–F) LINK-A increased Bcl2 expression by activating AKT signaling.

Previous studies have demonstrated that LINK-A over-activates AKT signaling in MCL (Lin et al., 2017b), therefore we next investigated whether LINK-A increases Bcl2 expression by activating AKT. Figure 4A showed that the activation of AKT was higher in Ibrutinib-resistant MAVER-1 cells than in Ibrutinib-sensitive Jeko-1 cells, and the Bcl2 expression was increased MAVER-1 cells compared with Jeko-1 cells. LINK-A overexpression resulted in the AKT activation in Jeko-1 cells, whereas knockdown of LINK-A repressed AKT activation in MAVER-1 cells (Figs. 4B and 4C). More important, AKT inhibitor MK2206 repressed LINK-A-induced increase of p-AKT activation and Bcl2 expression (Fig. S2, Fig. 4D), and resulted in subsequent decrease of cell viability and increase of cell apoptosis in the presence of Ibrutinib and LINK-A (Figs. 4E and 4F).

## Discussion

In the current study, the role of LINK-A lncRNA in intrinsic Ibrutinib resistance in MCL was verified and its underlying mechanism was identified. The present results demonstrate that (i) LINK-A expression was elevated in Ibrutinib-resistant MCL cell lines; (ii) LINK-A enhanced cell viability and repressed Ibrutinib-induced cell apoptosis; (iii) LINK-A enhanced Bcl2 expression in the presence of Ibrutinib; (iv) LINK-A increased Bcl2 expression by activating AKT signaling. These results uncovered the important role of LINK-A inhibition in overcoming intrinsic Ibrutinib resistance by regulating the AKT/Bcl2-mediated anti-apoptosis pathway, and might provide a therapeutic opportunity for Ibrutinib-resistant MCL patients.

LINK-A, a newly identified lncRNA, exerts oncogenic functions in different types of tumors, including breast cancer (Lin et al., 2017b; Lin et al., 2016b), glioma (Hua et al., 2019), metastatic osteosarcoma (Zhao, Liu & Cai, 2019), Non-small-cell lung cancer (Zhao et al., 2018), and MCL (Zhang et al., 2019). Lin et al. demonstrated that LINK-A promotes normoxic hypoxia inducible factor-1*α* (HIF-1*α*) signaling activation in triple-negative breast cancer (TNBC) by recruiting BRK to the EGFR:GPNMB complex and thus to activate BRK (Lin et al., 2016b). LINK-A promotes AKT inhibitors resistance through over-activating AKT signaling in breast cancer cells (Lin et al., 2017b). Recently, the role of LINK-A in MCL has also been verified. Zhang et al. (2019) reported that up-regulated LINK-A accelerates MCL cells proliferation and represses cell apoptosis through increasing Survivin expression. Given the role of LINK-A in down-regulating cancer cell antigen presentation and intrinsic tumor suppression, we speculated that LINK-A might be correlated with intrinsic ibrutinib resistance in MCL cells.

Gene expression profiling analysis of MCL cells identifies the abnormality of many proteins involved in AKT signaling, which is hyper-activated in MCL cells (Rizzatti et al., 2005; Dal Col et al., 2008). Dal Col et al. (2012) demonstrated that AKT is over-activated in MCL, and pharmacologic inhibition of Akt enhances the expression of pro-apoptotic BH3-only Noxa protein and represses the expression of anti-apoptotic Bcl-xL and Bfl-1 proteins. In the present study, we found that AKT activation is higher in Ibrutinib-resistant MCL cells than in Ibrutinib-sensitive cells. Forced expression of LINK-A promotes AKT signaling activation in Jeko-1 cells, whereas LINK-A knockdown suppresses AKT activation in MAVER-1 cells. Especially, AKT inhibition facilitates cell apoptosis in the presence of Ibrutinib and LINK-A, indicating that LINK-A contributes to Ibrutinib resistance of MCL cells, at least in part by regulating AKT-dependent target genes.

Based on the previous studies, in which aberrant Bcl2 expression is associated with BTK activation and MCL progression (Li et al., 2016; Agarwal et al., 2018), we thus investigated whether LINK-A regulates Bcl2 expression by the mediation of AKT. The current results showed that LINK-A overexpression increases Bcl2 expression, whereas LINK-A knockdown represses Bcl2 expression. Importantly, AKT inhibition destroys LINK-A-induced enhancement of Bcl2, and results in subsequent increase of cell apoptosis in the presence of Ibrutinib and LINK-A.

The limitations of the study are as follows: (i) The plasma level of LINK-A was not assessed in MCL patients before and after treatment. (ii) The plasma level of LINK-A was not assessed in Ibrutinib-resistant MCL patients. (iii) As a cytoplasmic lncRNA, LINK-A might function as a competing endogenous RNA (ceRNA) as other cytoplasmic lncRNAs, which absorbs indicated miRNAs and thus indirectly regulates mRNA expression. In the study, the LINK-A-miRNA-mRNA ceRNA network was not identified.

## Conclusion

The current data revealed the function of LINK-A inhibition on overcoming Ibrutinib resistance in MCL cells by regulating the AKT/Bcl2 pathway, and might provide a novel target for Ibrutinib-resistant MCL patients.

##  Supplemental Information

10.7717/peerj.12571/supp-1 Supplemental Information 1 Bcl2 overexpressin or knockdown

10.7717/peerj.12571/supp-2 Supplemental Information 2 LINA-A activated AKT signaling

10.7717/peerj.12571/supp-3 Supplemental Information 3 Apoptosis-related genes deregulated in MCL ^1^

10.7717/peerj.12571/supp-4 Supplemental Information 4 Raw data for Figs. 1 and 2

10.7717/peerj.12571/supp-5 Supplemental Information 5 Raw data for Figs. 3 and 4

10.7717/peerj.12571/supp-6 Supplemental Information 6 Raw data: WB

## Additional Information and Declarations

### Data Availability

The following information was supplied regarding data availability: The raw measurements are available in the Supplementary File.
