# Supplementary figures and images for: Inhibition of LINK-A lncRNA overcomes ibrutinib resistance in mantle cell lymphoma by regulating Akt/Bcl2 pathway
Source: PeerJ. 2021 Dec 17;9:e12571. doi: 10.7717/peerj.12571 (PMC8686732; doi:10.7717/peerj.12571)

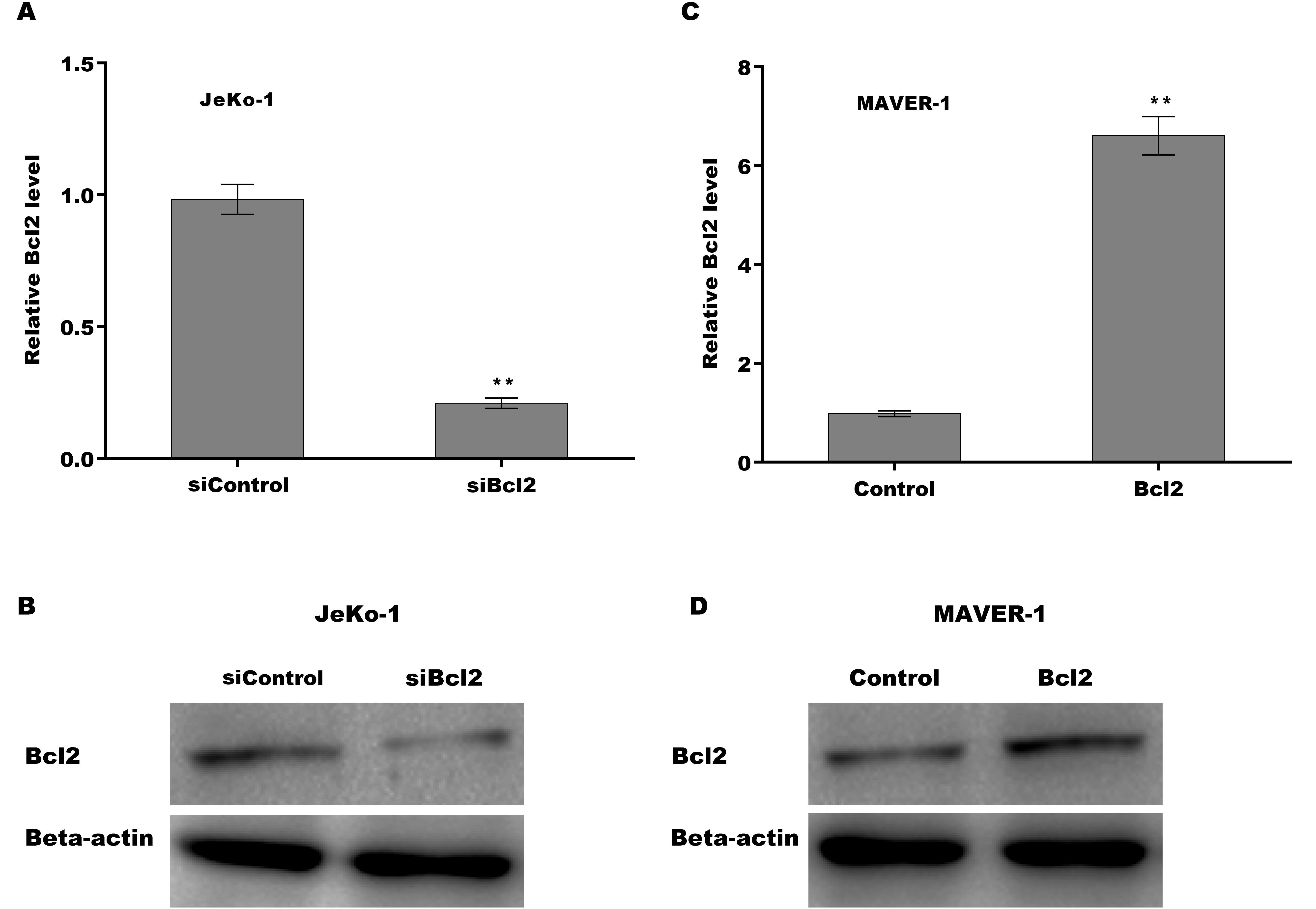

Supplement: Supplemental Information 1 [file peerj-09-12571-s001.png]

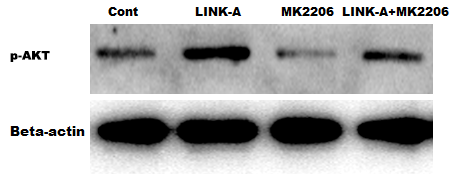

Supplement: Supplemental Information 2 [file peerj-09-12571-s002.png]

Figure 3

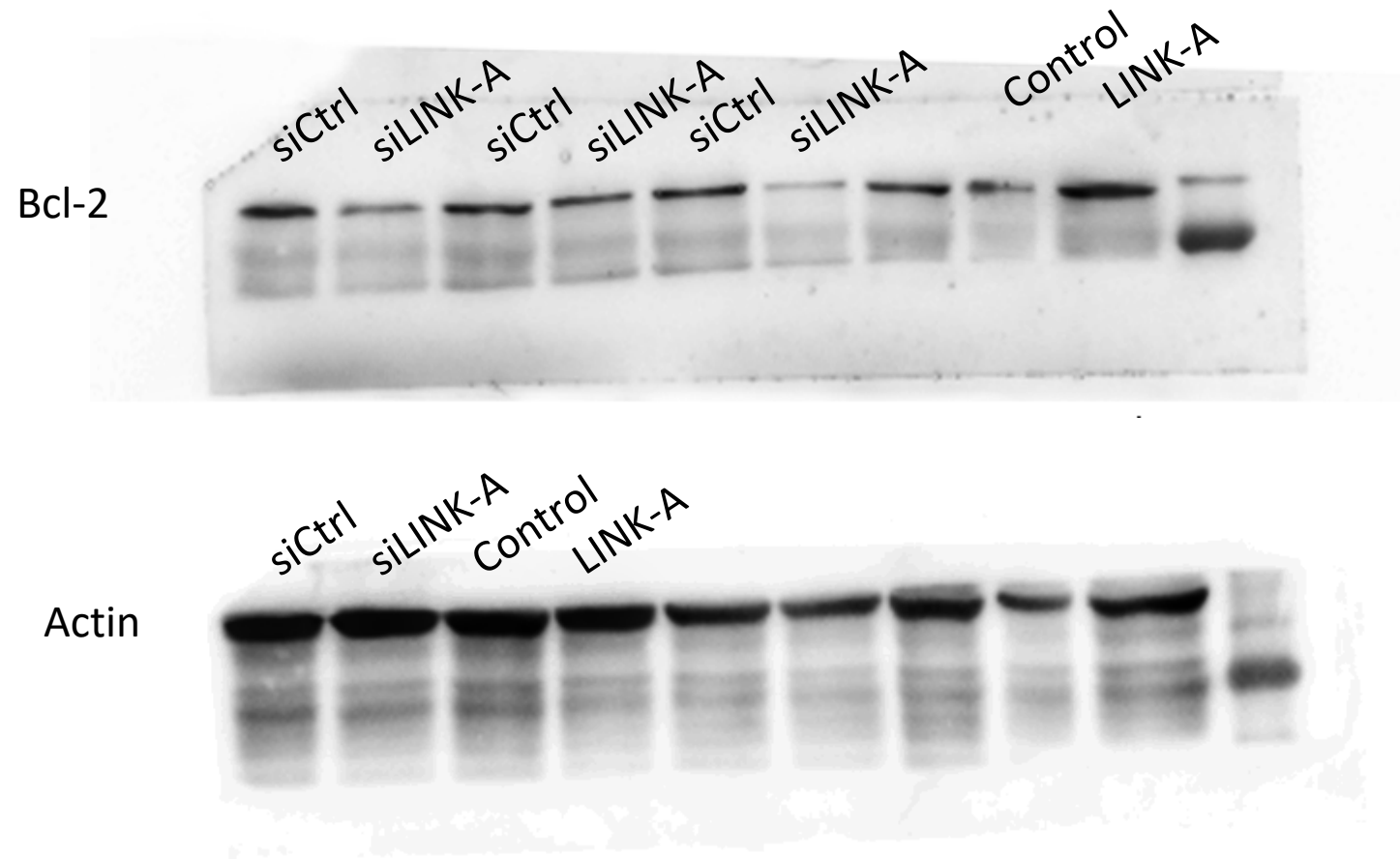

Figure 4

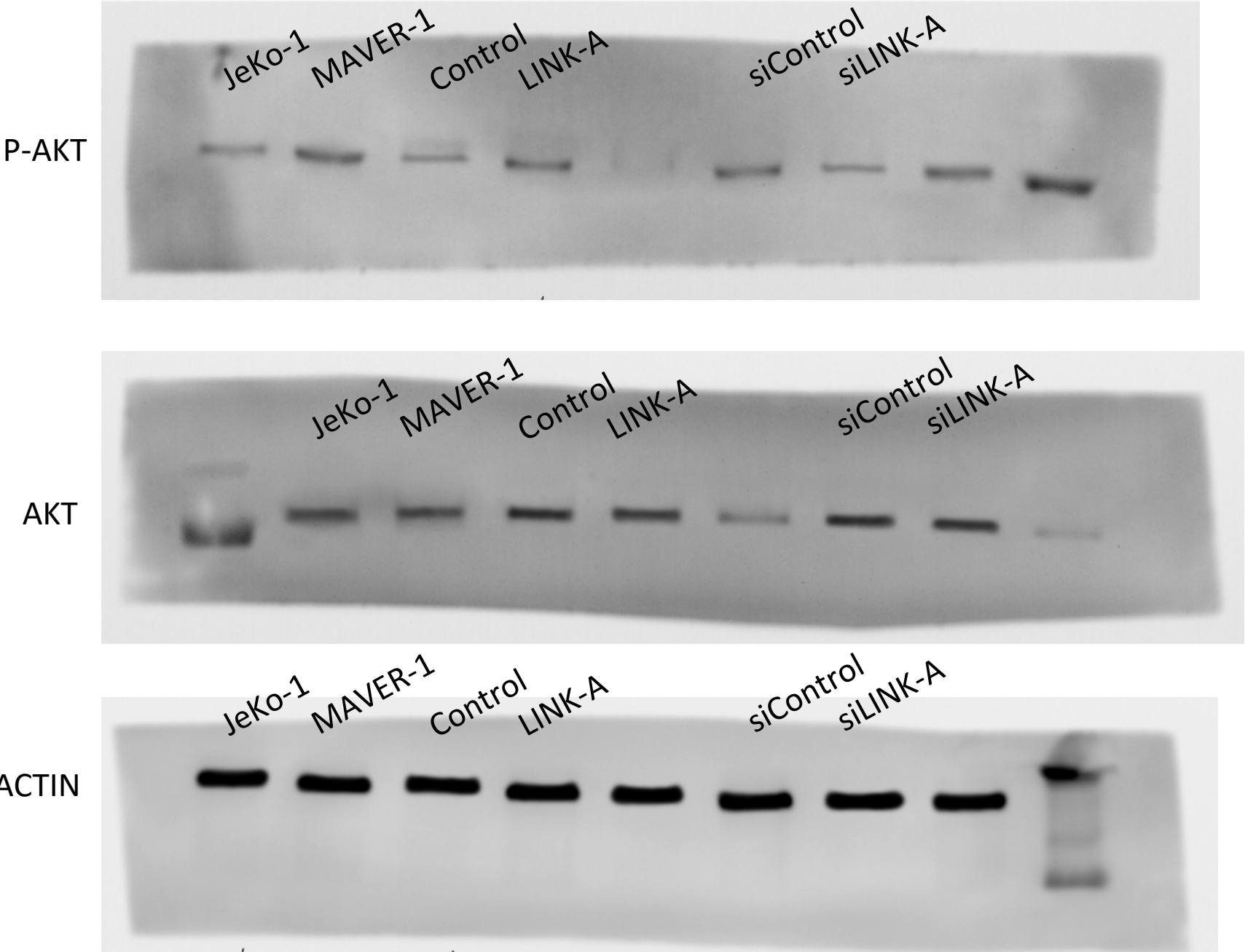

Supplement: Supplemental Information 6 [file peerj-09-12571-s006.pdf]
