# Supplementary material for: Inhibition of LINK-A lncRNA overcomes ibrutinib resistance in mantle cell lymphoma by regulating Akt/Bcl2 pathway
Source: PeerJ. 2021 Dec 17;9:e12571. doi: 10.7717/peerj.12571 (PMC8686732; doi:10.7717/peerj.12571)
Supplement: Supplemental Information 3 [file peerj-09-12571-s003.docx]

**Supporting Table S1. Apoptosis-related genes deregulated in MCL**[**^1^**](#_ENREF_1)

| **Gene symbol** | **Gene description** | ***n* fold (average)** | Upregulated |
| --- | --- | --- | --- |
| BCL2 | B-cell CLL/lymphoma2 | 5.855 |  |
| TOSO | Regulator of Fas-induced apoptosis | 4.588 |  |
| PIK3CD | Phosphoinositide-3-kinase, catalytic, δ polypeptide | 3.525 |  |
| IKBKB | Inhibitor of κ light polypeptide gene enhancer in B-cells, kinase β | 3.259 |  |
| TNFRSF7 | Tumor necrosis factor receptor superfamily, member 7 | 2.995 |  |
| TNFSF4 | Tumor necrosis factor(ligand) superfamily, member 4 | 2.878 |  |
| TRAF5 | TNF receptor-associated, factor 5 | 2.814 |  |
| FIP2 | Tumor necrosis factor (ligand) superfamily, member 13 | 2.675 |  |
| TNFSF13 | Lymphotoxin β (TNF superfamily, member3) | 2.420 |  |
| LTB | TRAF family member-associated NFκB activator | 2.410 |  |
| TANK | TNF receptor-associated factor 6 | 2.325 |  |
| TRAF6 | Apoptosis-associated speck-like protein containing a CARD | 2.321 |  |
| ASC | CASP8 and FADD-like apoptosis regulator | 2.250 |  |
| CFLAR | Apoptosis-antagonizing transcription factor | 2.152 |  |
| DED | Tumor necrosis factor, α-induced protein 3 | 2.111 |  |
| TNFAIP3 | Caspase1, apoptosis-related cysteine protease | 2.075 |  |
| CASP1 | Caspase7, apoptosis-related cysteine protease | 0.464 | Downregulated |
| CASP7 | Serine/threonine kinase17a (apoptosis-inducing) | 0.422 |  |
| STK17A | Chromosome segregation1 (yeast homolog)-like | 0.406 |  |
| CSE1L | Baculoviral IAP repeat-containing2 | 0.394 |  |
| BIRC2 | Serine/threonine kinase 17b (apoptosis-inducing) | 0.388 |  |
| STK17B | Baculoviral IAP repeat-containing5 (survivin) | 0.360 |  |

1. Martinez, N., Camacho, F. I., Algara, P., Rodriguez, A., Dopazo, A., Ruiz-Ballesteros, E., Martin, P., Martinez-Climent, J. A., Garcia-Conde, J., Menarguez, J., Solano, F., Mollejo, M. & Piris, M. A. (2003). The molecular signature of mantle cell lymphoma reveals multiple signals favoring cell survival. Cancer research 63, 2003.
